# Supplementary material for: Iron whiskers on asteroid Itokawa indicate sulfide destruction by space weathering
Source: Nat Commun. 2020 Feb 28;11:1117. doi: 10.1038/s41467-020-14758-3 (PMC7048718; doi:10.1038/s41467-020-14758-3)
Supplement: Supplementary file 1 — Supplementary Information [file 41467_2020_14758_MOESM1_ESM.pdf]

Supplementary Information for

Iron whiskers on asteroid Itokawa indicate sulphide destruction by space weathering

Matsumoto et al.

Contents:

Supplementary Fig. 1: FIB sections RA-QD02-00325-01 and RA-QD02-0325-02

Supplementary Fig. 2: FIB section RA-QD02-0292-01

Supplementary Fig. 3: FIB section RA-QD02-0286-0.

Supplementary Fig. 4: Outermost vapor-deposited rim and polynanocrystalline rim of olivine coexisting with troilite

Supplementary Fig. 5: Solar flare tracks in olivine coexisting with troilite

Supplementary Fig. 6: Calculated depth profiles of 1keV H and 4keV He implanted into troilite

Supplementary Fig. 7: Estimation of sulphur evaporation in a vesicle with H_2 gas.

Supplementary Fig. 8: Calculation of the amount of excess iron by SEM images

Supplementary Fig. 9: Diffusion length of iron in troilite per year against surface temperature on Itokawa.

Supplementary Table 1: Itokawa particles investigated in this study

Supplementary Table 2: TEM-EDX elemental compositions of whiskers

Supplementary Table 3: Troilite depths and whisker volumes

Supplementary Note 1: Classification of iron sulphide surface of Itokawa grains

Supplementary Note 2: Troilite in observed FIB sections RA-QD02-0325-01, RA-QD02-0292-01, and RA-QD02-0286-01

Supplementary Note 3: Possibility of iron whiskers being artifacts during sample preservation

Supplementary Note 4: Special attributes of observed Itokawa grains

Supplementary Note 5: Removal of sulphur by the chemical reaction between troilite and solar wind H_2 gas

Supplementary Note 6: Estimation of the sulphur evaporation rate by solar heating (H-free environment)

Supplementary Note 7: Calculation of the amount of excess iron atoms on troilite surface

Supplementary Note 8: Calculation of iron diffusion in troilite

Supplementary References

Supplementary Figures

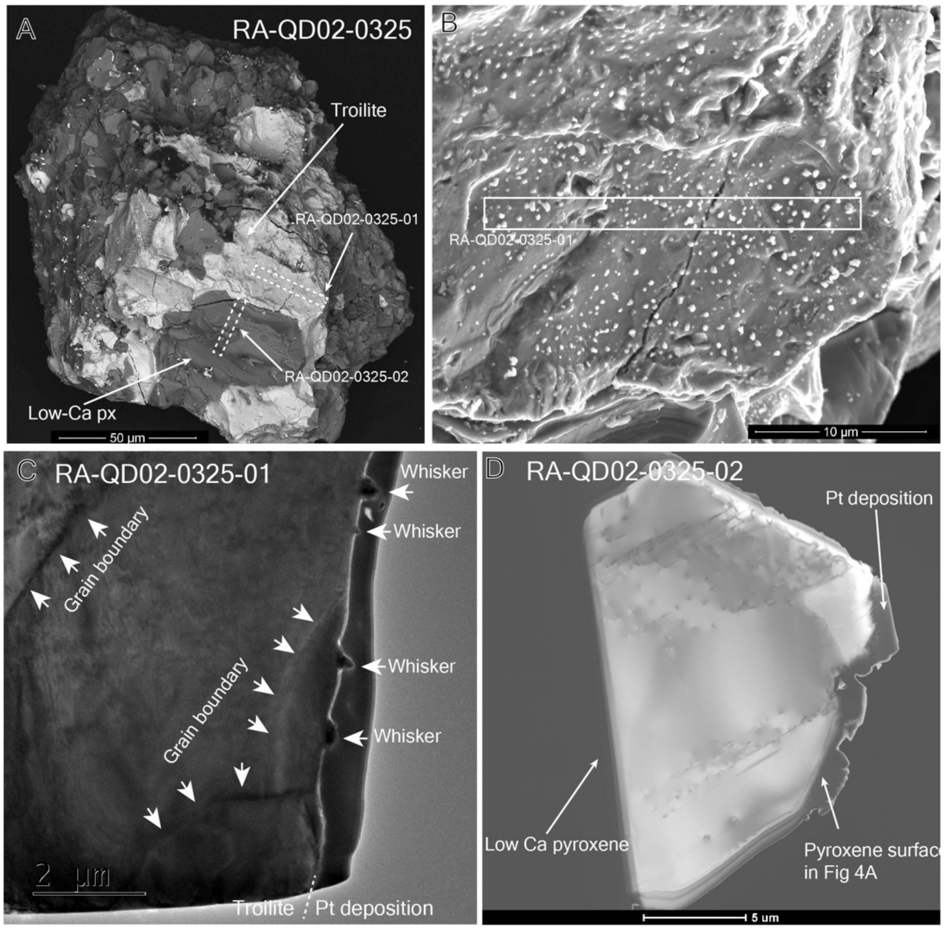

Supplementary Fig. 1. **FIB sections RA-QD02-00325-01 and RA-QD02-0325-02.** (A) A backscattered electron (BSE) image of RA-QD02-0325. RA-QD02-0325 is composed of olivine, low-Ca pyroxene, plagioclase, and iron sulphide. Troilite grains (bright regions) that are > 10 μm in size are dispersed on the grain surfaces. FIB sections of troilite (RA-QD02-00325-01) and pyroxene (RA-QD02-0325-02) were made from the areas of the dotted squares. (B) An enlarged secondary electron (SE) image of troilite where the FIB section was lifted out (indicated by a white square). Numerous whiskers can be identified as bright spots on the troilite. (C) A TEM bright-field (BF) image of RA-QD02-0325-01. RA-QD02-0325-01 is composed of polycrystalline troilite. In this section, seven whiskers are present on the troilite grain surface.

(D) A low-angle annular dark-field (LAADF)-STEM image of RA-QD02-0325-02, which is composed of low-Ca pyroxene.

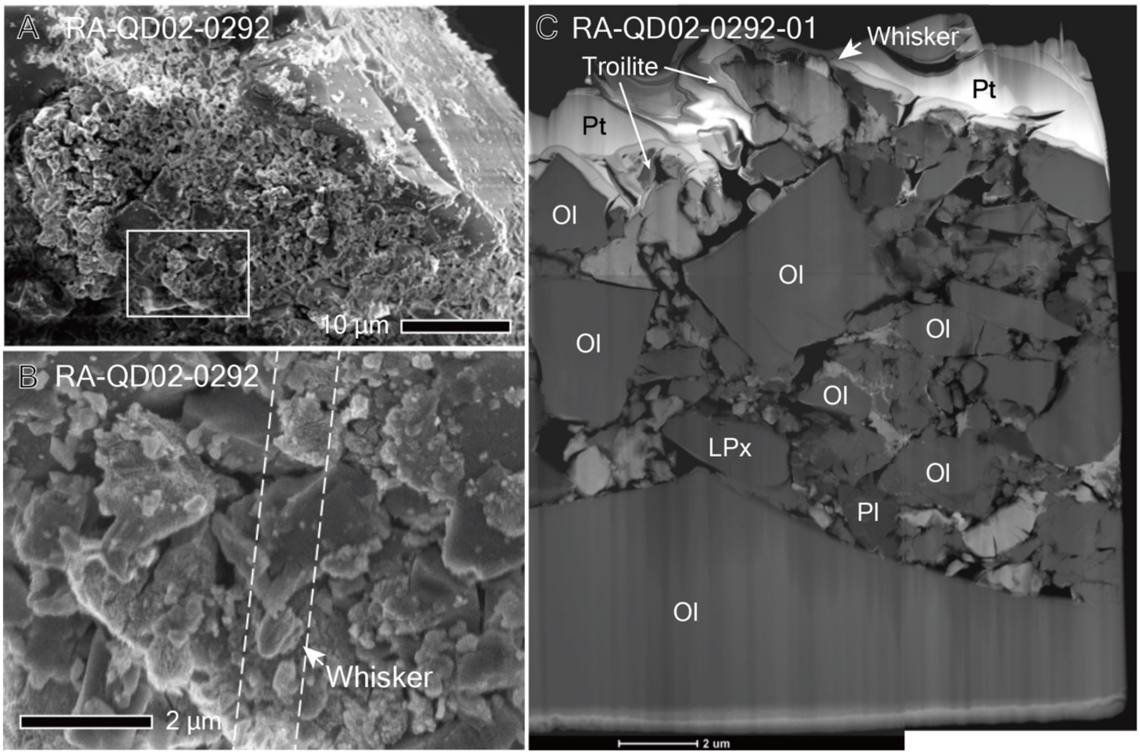

Supplementary Fig. 2. FIB section RA-QD02-0292-01. (A)(B) SE images of RA-QD02-0292. The host olivine surface is partly covered with fine fragments of olivine, pyroxene, plagioclase, and iron sulphide. Section RA-QD02-0292-01 was made in the area between the dotted lines. (C) A BF-STEM image of RA-QD02-0292-01. This section comprises the host olivine and numerous mineral fragments up to 5 μm in size aggregated on the host olivine. Troilite fragments are on the top of the fine aggregates. An iron whisker marked with an arrow corresponds to the whisker in Fig. 1. Ol = olivine, Pl = plagioclase, LPx = low Ca pyroxene.

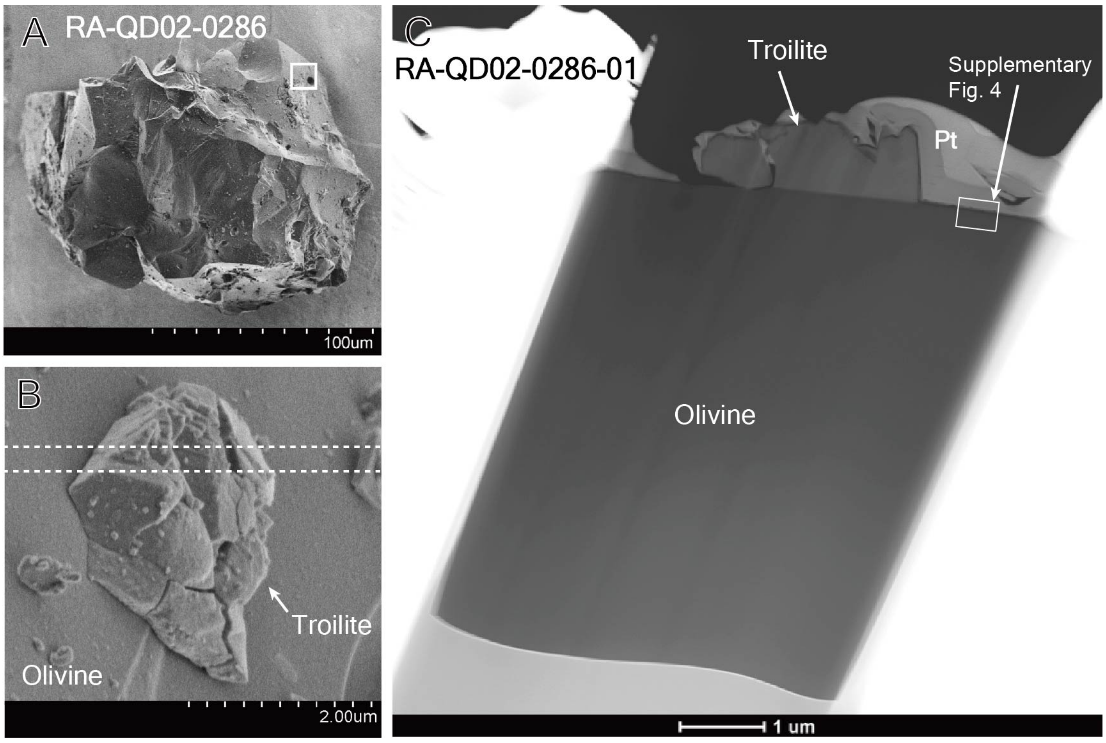

Supplementary Fig. 3. **A FIB section RA-QD02-0286-01.** (A) A secondary electron image of RA-QD02-0286. The white square corresponds to (B). (B) An enlarged image of troilite on olivine surface. Section RA-QD02-0286-01 was made in the area between the dotted lines. (C) A BF-STEM image of RA-QD02-0286-01. Troilite is located on a large host olivine.

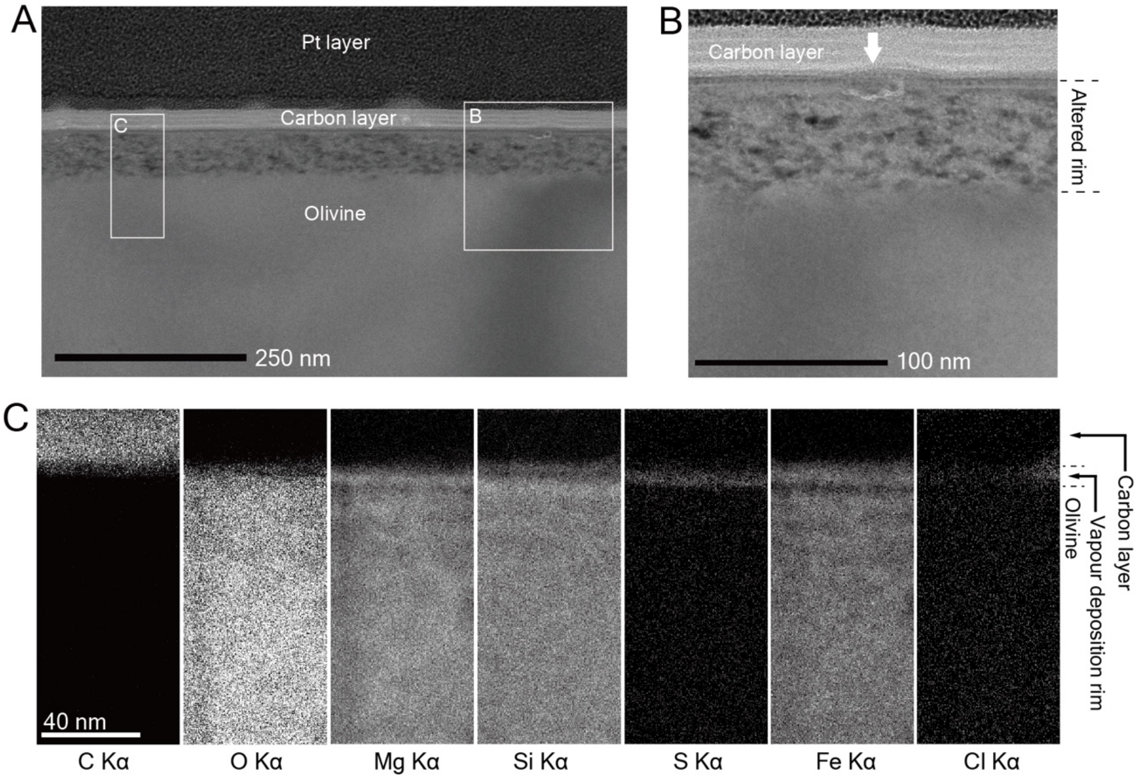

Supplementary Fig. 4. Outermost vapor-deposited rim and polynanocrystalline rim of olivine coexisting with troilite. (A) TEM-BF image of altered rims of olivine in FIB section RA-QD02-0286-01. This image was obtained from a rectanglur region shown in Supplementary Fig. 3C. The olivine surface was coated by carbon and Pt layers for SEM/FIB work. (B) Enlarged TEM-BF image of the olivine surface. The altered rim shows patchy contrast due to crystallographic misorientation in the rim, whereas the unaltered region beneath the rim has uniform contrast. A possible vesicle is marked with an arrow. (C) The X-ray intensity maps of major elements detected by EDX analysis of the olivine. The vapor deposition rim corresponds to the layer including elements sulphur and chlorine which are not contained in the unaltered olivine.

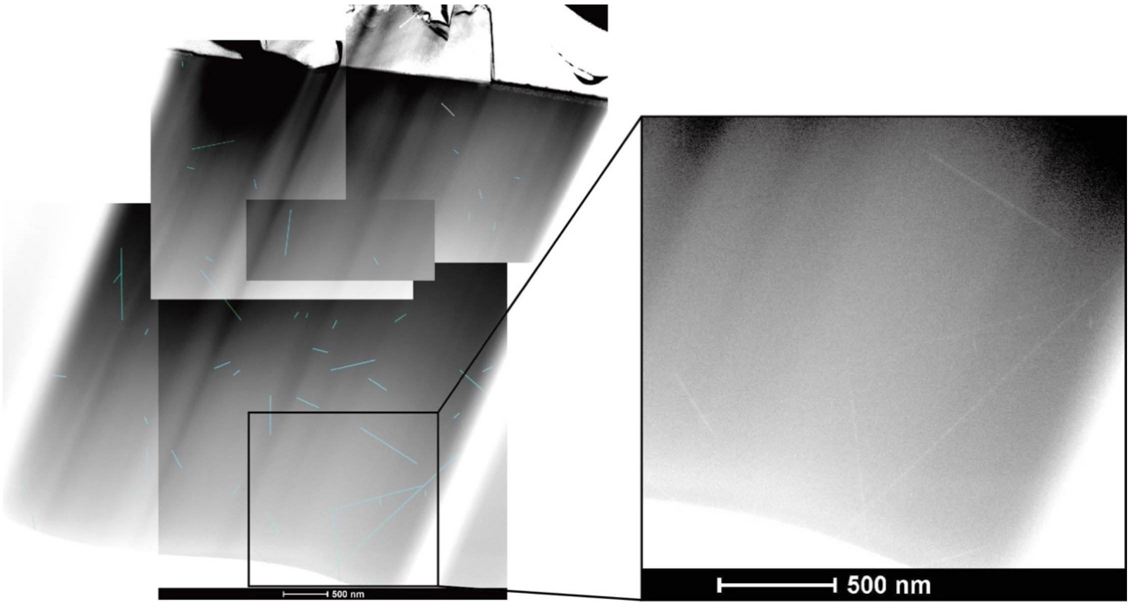

Supplementary Fig. 5. Solar flare tracks in olivine coexisting with troilite. A low-angle annular dark field (LAADF)-STEM image montage of RA-QD02-0286-01. Solar flare tracks are indicated by the blue lines. The enlarged image on the right is a raw image. Estimated track densities in the observed FIB sections are listed in Supplementary Table 1.

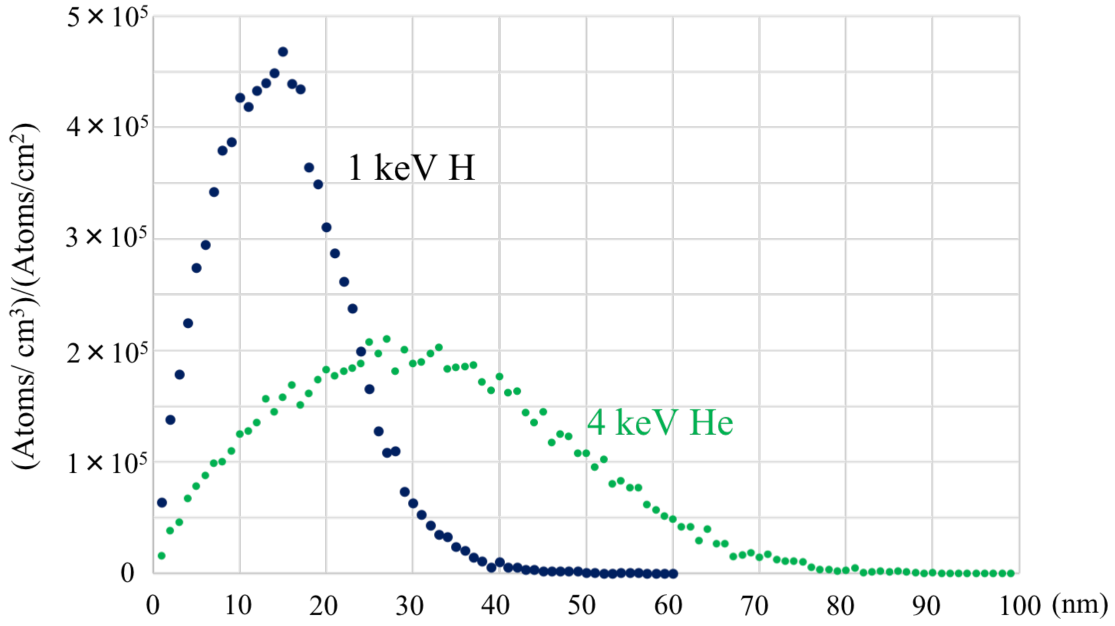

Supplementary Fig. 6. Calculated depth profiles of 1keV H and 4keV He implanted into troilite. The atomic distribution of 1keVH and 4keV He implanted into troilite were calculated using a Monte-Carlo simulation in SRIM[26]. Vertical axis is ion concentration, and lateral axis is the depth from the troilite surface. The ion penetration depth defined as the sum of the average projected range and the longitudinal straggle is ~22 nm and ~47 nm for 1 keV hydrogen and 4 keV helium, respectively.

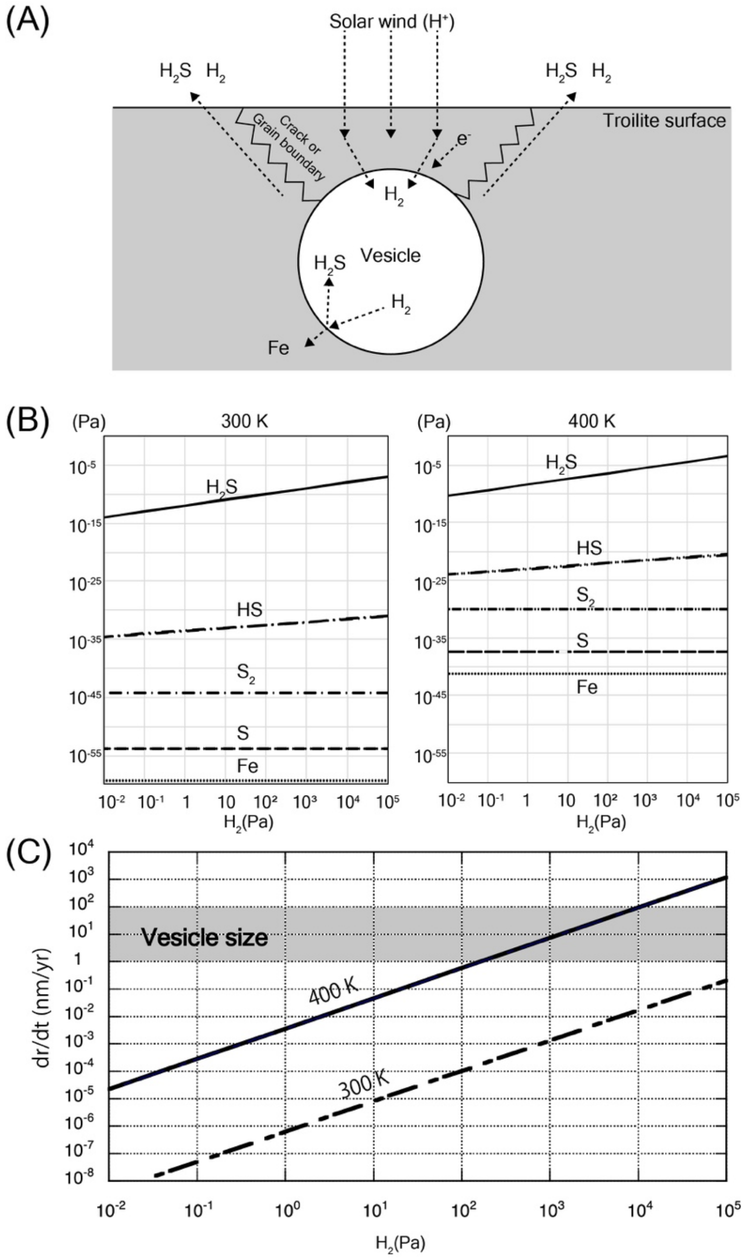

Supplementary Fig. 7. **Estimation of sulphur evaporation in a vesicle with H_2 gas.** (A) A schematic image of a simple vesicle model with constant supply of H_2 gas and escape of gaseous products. (B) Estimated vapor pressure of S-bearing species and Fe gas on troilite plotted against H_2 pressure at 300 K (left) and 400 K (right). (C) Calculated vesicle growth rate at 300 K and 400 K. The size range of vesicles observed in Itokawa grains (a few nm up to 100 nm) is shown by the grey bar.

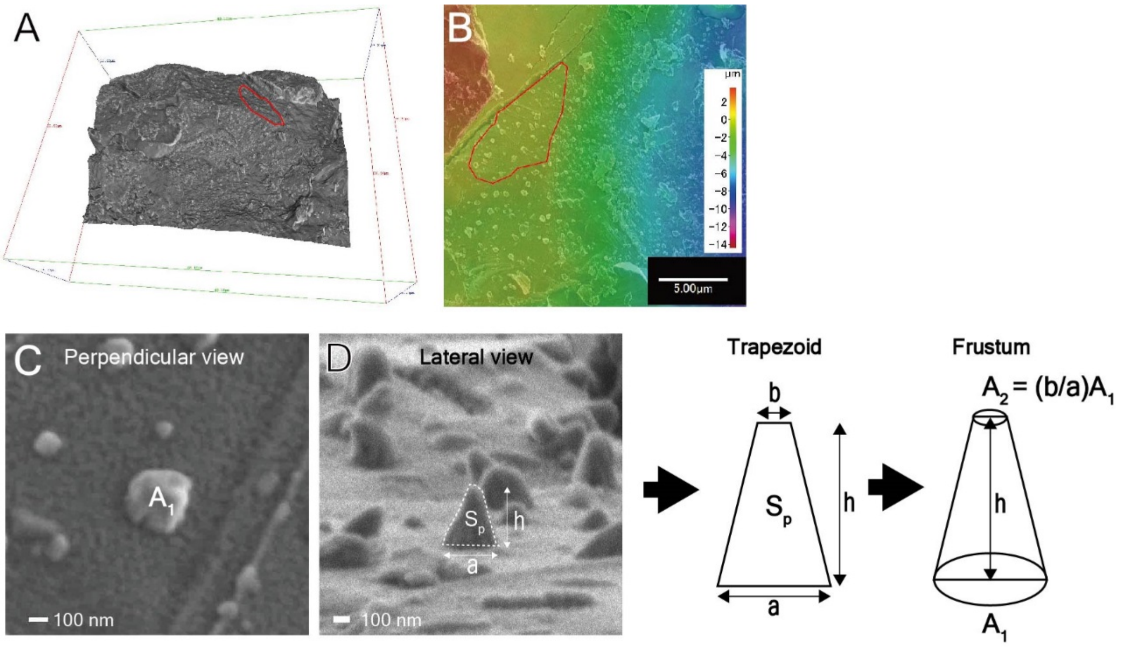

Supplementary Fig. 8. **Calculation of the amount of excess iron by SEM images**. (A) A 3-D model of the troilite surface constructed using stereo pairs of secondary electron images. One investigated area is surrounded by a red line. (B) A 3-D model viewed from perpendicular to the investigated area (shown by a red line). The coloured scale corresponds to relative height. (C) A secondary electron image of an iron whisker viewed from above. (D) A secondary electron image of an iron whisker viewed from the side and schematic images of the approximations used to calculate whisker volume.

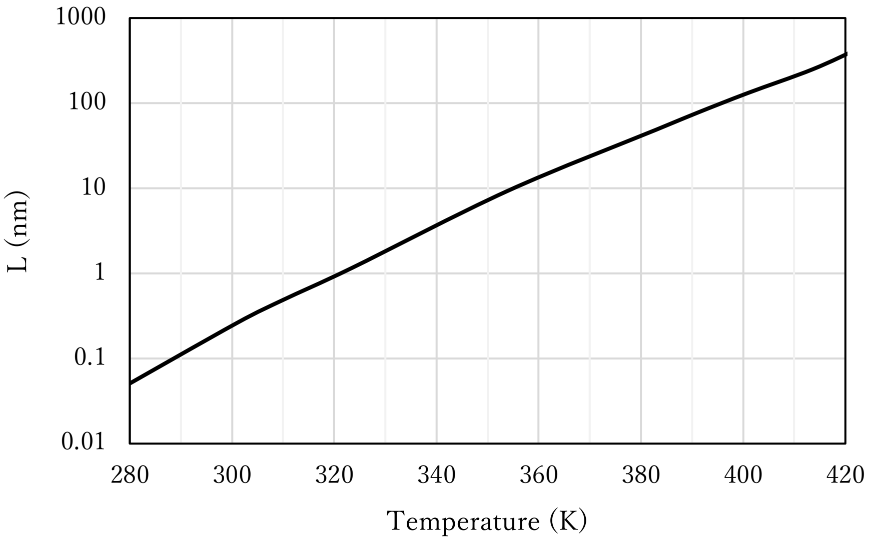

Supplementary Fig. 9. **Diffusion length of iron in troilite (L) per year against surface temperature on Itokawa.**

Supplementary Tables

Supplementary Table 1. Itokawa particles investigated in this study.

Group[a]	ID	Mineral phase[b]	Diameter (μm)[c]	Appearance of iron sulfide[d]	Whisker (max. length)[e]	Cavities[e]	Plates[e]	SFT density ($\times 10^8$ tracks/cm^2)[f]	Resident time (year)
I	RA-QD02-0282	ol,chm	241	S / Fine fragments	< 1.2 μm	i	nf	-	-
	RA-QD02-0292	ol, pl,lpx,tr	78	Fine fragments / S	< 1.3 μm	i	nf	3.3 ±0.2 (Olivine)	8×10^3
	RA-QD02-0302	tr,lpx	105	L*	< 1.3 μm	i	i	-	-
	RA-QD02-0325	FeS,ol,pl,lpx	166	L	< 2.0 μm	i	i	0.34 to 0.84 (Pyroxene)	1×10^3
II	RA-QD02-0275	ol	122	Melt splash	< 0.1 μm	nf	nf	-	-
	RA-QD02-0286	pl,ol,hpx,tr	168	S	< 0.6 μm	nf	nf	1.7 ±0.3 (Olivine)	4×10^3
	RA-QD02-0301	tr,pl,ol	213	L	< 0.5 μm	nf	i	-	-
	RA-QD02-0331	FeS,pl,lpx,ol,	148	L*	< 0.2 μm	nf	i	-	-
III	RA-QD02-0277	hpx,lpx,ol,pl,tr.chm	171	S	nf	nf	nf	-	-
	RA-QD02-0296	pl,tr	68	L*	nf	nf	nf	-	-

Notes: [a] I : particles having cavities and whiskers. II : particles in which whiskers appear but cavity is not identified. III : particles without cavity and whiskers. [b]Mineral phases identified on grain surfaces by SEM observation.ol: olivine; hpx: high-Ca pyroxene; lpx: low-Ca pyroxene; pl: plagioclase; K-feld: potassium-dominant feldspars; tr: troilite; FeNi: iron-nickel metal; chm: chromite; ap: apatite. [c] Average values of long and short axes of Itokawa particles measured from SEM observation. The mineral phase and size of particles in this table are reported in detail in the Hayabusa sample list databese (http://hayabusaao.isas.jaxa.jp). [d]L = iron sulfide comprising a large poriton of Itokawa grain surface; S = iron sulfide comprising a small poriton of Itokawa grain surface. Asterisks indicate the appearance of euheral crystals. [e] i = identified; nf = not found. [f]The residence time of Itokawa grains within a few millimeters from the uppermost regolith surface were estimated based upon track production rate for a 2π exposure of resulting in 4.1×10^4 tracks/cm^2/yr (Keller et al., 2015[23]).

Supplementary Table 2. TEM-EDX elemental compositions of whiskers.

Sample	Whisker	Cr wt%	Fe wt%	Co wt%	Ni wt%	Comment
RA-QD02-0286-01	1*	<0.5	99.8	<7.0	<0.7	Whole whisker
RA-QD02-0292-02	1*	<0.2	95.9	<3.0	4.1	Most Ni-rich area
RA-QD02-0292-02	1*	<0.1	98.1	<1.0	1.9	Ni-rich area
RA-QD02-0292-02	1*	<0.1	98.9	<1.0	1.1	Whole whisker
RA-QD02-0325-01	1	<0.1	99.7	<2.0	0.2	Whole whisker
RA-QD02-0325-01	1	<0.5	99.9	<7.0	<0.7	Center
RA-QD02-0325-01	1	<0.2	99.8	<3.0	<0.3	Tip
RA-QD02-0325-01	6	<0.1	99.7	<2.0	<0.2	Whole whisker
RA-QD02-0325-01	6	<0.3	99.8	<4.0	<0.4	Tip
RA-QD02-0325-01	7	<0.4	99.8	<6.0	<0.6	Center

*These FIB sections only contained one whisker. Detection limits vary due to counting statistics.

Supplementary Table 3. Troilite depths and whisker volumes.

	Surface area (10^{-7} cm^2)	Number of whiskers	Q (10^{17} atoms/cm^2)	Corresponding depth of troilite (nm)	Corresponding depth of troilite (monolayer)
area 1	3.8	39	1.9	56	194
area 2	2.2	11	1.1	32	109
area 3	3.2	24	0.9	26	90
Average	3.1	25	1.3	38	131

Supplementary Information

Supplementary Notes

Supplementary Note 1: Classification of iron sulphide surface of Itokawa grains.

The features observed on the various iron sulphide surfaces are further summarized in Supplementary Table 1. Itokawa particles can be divided into three groups based on the appearance of whiskers and cavities on iron sulphides. Particles with cavities and whiskers are classified into a first group. The second group comprises particles on which whiskers are present, but cavities were not identified. The third group consists of particles having cavity-free surfaces and no whisker. The lengths of the whiskers on the cavity surfaces ranges between ~ 1.3 µm and 2.8 µm. They are significantly longer than the whiskers on the cavity-free surfaces, which ranges between 200 nm and 550 nm in length. Blisters and small impact craters are also found on silicate surfaces of the investigated grains (fig. 2F in Matsumoto et al. 2018[51]).

Supplementary Note 2 :Troilite in observed FIB sections RA-QD02-0325-01, RA-QD02-0292-01, and RA-QD02-0286-01.

Iron sulphides in these sections were identified as troilite from selected area electron diffraction (SAED) patterns, based on characteristic superstructure reflections (Fig. 3B, D) and EDX analysis. The SAED

pattern in Fig. 3D shows additional very weak and diffuse spots from a pyrrhotite-like superstructure, but pyrrhotite was not found as a discrete phase, suggesting that a disordered layer may be present at the section surface due to damage of the crystal lattice during FIB sectioning. Quantitative EDX analysis of the iron sulphides in the three sections was performed using FE-TEM (JEOL JEM-3200FSK) at Kyushu University (see Methods). Chemical compositions of the iron sulphides in the three sections are consistent with stoichiometric FeS, given the analytical 2σ error of ±3%.

Section RA-QD02-0325-01 was obtained from a large troilite grain that is ~50 µm in size and thus the whole FIB section consists of troilite. This troilite grain is a polycrystalline aggregate, and comprises troilite crystals that are a few microns in size (Supplementary Fig. 1).

Section RA-QD02-0292-01 was obtained from an area where fine fragments of iron sulphides that are ~2 µm in size are exposed at the grain surface (Supplementary Fig. 2). The section consists of a large olivine grain, which is partly covered with fine fragments of olivine, pyroxene, plagioclase, and iron sulphide. Section RA-QD02-0286-01 includes a troilite grain that is ~2.5 µm in width and 850 nm in thickness, which is located on a large olivine grain (Supplementary Fig. 3).

Supplementary Note 3: Possibility of iron whiskers being artifacts during sample preservation.

Since the reentry capsule of Hayabusa spacecraft was brought back to JAXA, Itokawa grains have been preserved in a clean chamber filled with purified nitrogen gas which contains impurities of less than

1ppmV[66]. Until our FE-SEM observation (Fig. 1), Itokawa grains had directly touched only pure aluminum-coated walls of the sample catcher, quartz glass disks for sample storage, quartz glass probes of manipulation devices, and gold-coated copper disks in a holder for SEM observation. Thus, Itokawa grains had never been in contact with any materials reactive towards troilite and had never been exposed to a reactive gas. Hence, it is highly unlikely that iron whiskers have formed during sample preservation.

Supplementary Note 4: Special attributes of observed Itokawa grains.

Iron whiskers have not been recognized in the previous studies. However, the observation of iron sulphides has been quite limited so far. The modal abundance of troilite is estimated to be low (2 %) by the preliminary examination of 40 Itokawa grains[19]. Space-exposed iron sulphides observed in prior studies are minor inclusions surrounded by silicate grains[14–16]. Moreover, observable iron sulphides were not identified in a previous systematical surface observation of 26 Itokawa grains[12]. Thus, it is reasonable that researchers in the previous studies were not able to encounter the space exposed iron sulphides with iron whiskers. This situation had been changed over a few years due to continuous effort of curatorial works in JAXA. Relatively large Itokawa grains (> 150 μm) had been collected from the sample catcher and some of them included iron sulphides as a major phase. The accumulation of available samples enabled us to describe general features of iron sulphides. We performed FE-SEM observations of Itokawa grains randomly recovered during the curatorial work. Because the observed grains appear to have similar compositional,

structural, and morphological features compared to other grains, it is likely that they are typical Itokawa grains. Thus, we rule out that Itokawa grains with whiskers were biased during sample selection or sampling on the asteroids. Therefore, surface features examined in our study may be representative of the entire Itokawa samples.

Supplementary Note 5: Removal of sulphur by the chemical reaction between troilite and solar wind H_2 gas

Sulphur evaporation may occur when troilite comes into contact with H_2 gas trapped in vesicles beneath the troilite surface. The H_2 gas may cause the chemical reaction with troilite under solar heating condition (aphelion: ~300K, perihelion: ~400K). Here, we determined the major chemical reaction and dominant factors for controlling the sulphur escape from the troilite surface. We developed a simple model in which gases in a vesicle are efficiently replaced by constant supply of H_2 gas from the solar wind and by escape of gases through cracks, grain boundaries and/or vesicle openings in the damaged rim (Supplementary Fig. 7A). This condition simulates a saturated state of solar wind accumulation to regolith, in which incoming and outgoing gaseous flux are almost equal. In an ideal condition, we assumed constant H_2 partial pressure in the vesicle due to continuous H_2 supply. The partial pressures of gaseous reaction products were assumed to be zero, simulating their efficient escape from the vesicle. The sulphur evaporation flux $J_i(S)$ of a sulphur-bearing gas species i from the vesicle wall can be estimated using the

Hertz-Knudsen-Langmuir equation with the equilibrium vapor pressures of sulphur-bearing species over troilite:

$$J_i(S) = \frac{\alpha_i[p_i^{eq} - p_i]}{\sqrt{2\pi m_i kT}} \tag{S1}$$

where α_i (≤ 1) is the evaporation coefficient, p_i^{eq}, the equilibrium pressure, p_i, the actual partial pressure, m_i, the molecular mass, k, the Boltzmann constant, T, the temperature. In our model, $p_i = 0$. The equilibrium vapor pressures of sulphur-bearing species can be calculated thermodynamically. Assuming the iron- troilite (IT) buffer, the major gas species in the system Fe-S-H are H_2, H_2S, HS, S, S_2, Fe, and the evaporation reactions are given as follows:

$$Fe(s) = Fe(g) + \frac{1}{2}S_2(g)$$

$$Fe(s) = Fe(g)$$

$$FeS(s) + \frac{1}{2}H_2(g) = Fe(g) + HS(g) \tag{S2}$$

$$HS(g) + \frac{1}{2}H_2(g) = H_2S\,(g)$$

$$S_2(g) = 2S(g)$$

The equilibrium constants for the above reactions are obtained from the Gibbs free energies of the reactions (NIST-JANAF, 1998):

$$K_1 = (p_{Fe}^{eq})(p_{S_2}^{eq})^{\frac{1}{2}}$$

$$K_2 = (p_{Fe}^{eq})$$

$$K_3 = (p_{Fe}^{eq})(p_{HS}^{eq})/(p_{H_2}^{eq})^{\frac{1}{2}} \tag{S3}$$

$$K_4 = (p_{H_2S}^{eq})/(p_{HS}^{eq})(p_{H_2}^{eq})^{\frac{1}{2}}$$

$$K_5 = \left(p_S^{eq}\right)^2/(p_{S_2}^{eq})$$

By solving Equations (S3) for given partial pressure of H_2, p_i^{eq} of the i gas can be obtained for 300K, and 400K (Supplementary Fig. 7B). Other gas species (H, S_3, S_4, S_5, S_6, S_7, and S_8) were ignored due to small equilibrium pressures. The equilibrium vapor pressure of H_2S is much larger than those of HS and S_2 at 300K and 400K (Supplementary Fig. 7B). Hence, the H_2 gas trapped in the vesicle may cause the reaction, $FeS + H_2 = H_2S + Fe^{2+} + 2e^-$ under solar heating condition. Thus, the total evaporation flux of sulphur can be expressed as follows:

$$J_{H_2S}(S) = \frac{\alpha[p_{H_2S}^{eq}]}{\sqrt{2\pi m_{H_2S}kT}} \tag{S4}$$

where the evaporation coefficient α is given by[47] $\alpha_{H_2S} = 2.03 \times 10^{-3} \left(p_{H_2}\right)^{0.106} \exp\left(-\frac{940}{T}\right)$, p_{H_2} is a partial H_2 pressure. When the vesicle size is ~10 nm, the Hertz-Knudsen-Langmuir equations can be adapted for the H_2 pressure below 10^5 Pa at 300 K to 400K. In the condition, Knudsen number of H_2 gas *Kn* satisfies $Kn > 10$, in which velocity distribution in the vapor is nearly Maxwellian. Here, the Knudsen number is defined as follows: $K_n = kT/(\sqrt{2}\pi\sigma^2 p_{H_2} L)$, where σ is molecular diameter of H_2 (0.3 nm), L is vesicle size. Using the equation S4, we estimated the growth rate of the vesicle, which is essentially promoted by sulphur evaporation. The increase of the vesicle volume due to evaporation of H_2S, ΔV, is expressed as follows: $\Delta V = 4\pi r^2 \Delta r = 4\pi r^2 \times J_{H_2S} \times v_{Fes} \times \Delta t$, where *r*, radius of the vesicle, Δr, the

increase of radius, t, time, v_s, a volume of a FeS molecule ($3 \times 10^{-29}\ m^3$). The growth rate of the vesicle is obtained from the relation:

$$\frac{dr}{dt} = J_{H_2S}(S) \times v_s. \tag{S5}$$

The growth rates (nm/yr) at 300K and 400 K are shown in Supplementary Fig. 7C. Assuming the fast diffusion of excess iron atoms into troilite, we ignored the appearance of iron metal residue that disturbs the sulphur evaporation. In our model, rapid sulphur evaporation and subsequent rapid vesicle growth occur under H_2 pressure above 10^3 Pa, compared with the size range of vesicles in Itokawa grains (a few nm – a few tens nm) and space exposure ages of the regolith (~10^3 yr). Previous H^+ ion irradiation experiments have estimated internal gas pressure of irradiation-produced vesicles to be up to 10^9 Pa[1]. Hence, hydrogen gas in troilite likely retained with pressures sufficient for the rapid vesicle growth. However, we assumed zero partial pressure of H_2S in this ideal model, simulating efficient escape of H_2S. When the gas replacement is ineffective, the evaporation flux of sulphur would slow down with increasing H_2S pressure (S1). Therefore, the sulphur evaporation may depend on the replacement rate of H_2 and H_2S in the vesicle. Additional heating events on Itokawa induced by micrometeorite bombardments and larger impact events may play a role for increasing diffusion rate of hydrogen atoms and H_2S in troilite, resulting in accelerating gaseous replacement rate. In addition, these heating events may raise the sulphur evaporation flux according to the temperature dependence of the equation S4.

Supplementary Note 6: Estimation of the sulphur evaporation rate by solar heating (H-free environment).

The reaction describing the sulphur evaporation by solar heating in a H-free environment is the oxidation of sulphide to S_2: $FeS + V_{Fe} = 0.5S_2 + Fe^{2+} + 2e^-$. If metallic iron precipitates, then the sulphur fugacity in equilibrium corresponds to the iron-troilite (IT) buffer. Depending on the temperature and the ambient sulphur fugacity near the surface of Itokawa, the reaction might be driven towards sulphur loss. The surface temperature of Itokawa may be up to 130 °C at the subsolar point assuming radiative equilibrium at 1 AU. At this temperature, the peak Maxwell-Boltzmann velocity of S_2 is about 323 m/s, which is much faster than Itokawa's escape velocity of 0.2 m/s and therefore S_2 may readily escape. This applies to practically any temperature that can be reasonably expected on Itokawa, and implies that the ambient S_2 partial pressure p would be far below the equilibrium value p^{eq}. Thus, a bare, space-exposed troilite surface would lose sulphur at a rate that is limited by internal diffusion, desorption, and the equilibrium vapor pressure. The Hertz-Knudsen-Langmuir relation (equation S1) allows estimation of the loss rate of S per unit area. At the IT buffer, the S_2 equilibrium pressure p^{eq} at 130°C is extremely small, and of the order of 10^{-29} Pa^2. Assuming an evaporation coefficient $\alpha = 1$ (although it is probably <1) and $p = 0$, a maximum evaporation flux of $\sim 3 \times 10^{-3}$ S atoms/cm^2/yr is obtained. This corresponds to $\sim 1 \times 10^{-18}$ nm/year, which is an exceedingly small rate. Heating within a porous regolith would probably further reduce sulphur loss by the build-up of some S_2 partial pressure due to the mean-free path of S_2

molecules being much larger than the pore radii.

Notably, a temperature increase in the uppermost regolith by 300 K would drastically increase the equilibrium partial pressure of S_2 and hence accelerate the thermally induced loss of sulphur from troilite, in the case of asteroids at low heliocentric distances where solar heating is more efficient. For the ideal case where the evaporation coefficient $\alpha = 1$ and $p = 0$, a recession of 40 nm per 10^3 years (corresponding to the estimated production rate of excess iron on Itokawa troilite; Supplementary Note 7) would be obtained if a perfectly permeable regolith is maintained at a temperature of ~433 °C (or slightly more if diurnal and seasonal variations, finite permeabilities, and small evaporation coefficients are taken into account). Sulphur loss increases exponentially with temperature, e.g., at 500 °C the rate of sulphur loss would amount to a recession of troilite surfaces of ~3 µm per 10^3 yr. A former orbit of Itokawa closer to the Sun appears possible, but to make heating the only mechanism for whisker growth it's surface temperature must have increased to above ~433 °C, which corresponds to an extreme orbital evolution[16] currently not backed by models. As the evaporation coefficient α is probably lower than unity under realistic kinetic conditions, solar heating alone might not have caused the whisker growth.

,

Supplementary Note 7: Calculation of the amount of excess iron atoms on troilite surface

We estimated the amount of excess iron atoms per unit area of troilite (Q) using the following equation;

$$Q = A_v d_{Fe} \left(\sum_i v_i\right) / m_{Fe} S_{FeS} \tag{S6}$$

where, A_v is the Avogadro constant, d_{Fe} is the density of metallic iron, m_{Fe} is the atomic mass of Fe, S_{FeS} is the investigated surface area of troilite, and v_i is the volume of a whisker located in the investigated area. The depth of troilite equal to the amount of excess iron can be calculated from $Q \times m_{FeS}/A_v d_{FeS}$, where m_{FeS} is the molar mass of troilite and d_{FeS} is the density of troilite. In order to calculate accurate S_{FeS} from SEM images, we constructed a three-dimensional (3-D) surface model from a stereo pair of secondary electron images (Supplementary Fig. 8AB). The pair of images was obtained by eccentric tilting of $\pm 5°$. The 3-D reconstruction was processed using Alicona's MeX software (www.alicona.co.uk). We approximated a whisker volume v_i as a frustum whose volume is given by $v_i = (A_1 + A_2 + \sqrt{A_1 A_2})\, h/3$, where A_1 and A_2 are the areas of the lower and the upper base respectively, and h is the height of the frustum. From a view almost perpendicular to (i.e., from above) the whiskers, we estimated A_1 from the basal area of a whisker (Supplementary Fig. 8C). From a lateral view (i.e., side), we estimated h from the height of a whisker (Supplementary Fig. 8D). For calculation of A_2, the projected area of the whisker (S_p) and basal length (a) from the lateral view were measured (Supplementary Fig. 8D). We assumed a trapezoidal shape with an area of S_p, the basal length a, and height h (Supplementary Fig. 8D). A_2 was estimated by assuming that $A_2 = A_1 \times (b/a)$, where b is the upper base of the trapezoid. We examined the whisker volume in three areas with relatively flat surfaces on RA-QD02-0325 (Supplementary Table. 3).

The average number of excess iron atoms was calculated to be 1.3×10^{17} atoms/cm^2. An equivalent

number of sulphur atoms should also have been removed from the troilite, at least. Considering the volume of one FeS molecule (~2.95 $\times 10^{-23}$cm^3/one FeS molecule: a half volume of the NiAs unit cell), the depth of troilite equal to the amount of excess iron bound in the whiskers is ~ 40 nm. This depth equals $\sim 1.3 \times 10^2$ monolayers of troilite, when a monolayer is defined as a S-S distance of 0.290 nm. The duration of exposure to the solar wind is estimated to be <1×10^3 yr, based upon solar ^{20}Ne concentrations in Itokawa particles[3]. The appearance of blisters on the silicate surfaces of RA-QD02-0325 indicates a relatively long duration of direct exposure to the solar wind. Considering this and the rather low SFT densities of RA-QD02-0325 we estimate that the duration of direct exposure of RA-QD02-0325 to the solar wind was $\sim 1 \times 10^3$ yr, during which the radiation-damaged rims and the SFTs were acquired simultaneously. Hence, the production rate of excess iron and the rate of sulphur loss are estimated to be ~0.04 nm/yr or ~0.13 troilite mono layers/yr. This is about ten orders of magnitude faster than thermally induced loss of S_2 at 130 °C.

Supplementary Note 8: Calculation of iron diffusion in troilite.

The characteristic length (L) of iron diffusion in troilite during the timescale of iron diffusion (t_d) can be estimated from $L^2 = t_d D_{tr}$, where D_{tr} is the self-diffusion coefficient of iron in troilite. D_{tr} was calculated using the diffusion coefficient D_{po} of iron in pyrrhotite ($Fe_{0.96}S$), which has been measured at temperatures > 443 K and depends on temperature and the ordering of Fe-site vacancies[4]. We used a diffusion coefficient

for iron in pyrrhotite (D_{Po}) given by the following equation[4];

$$D_{Po} = D_0 \exp\left[-\frac{Q_p(1+\alpha S(T)^2)}{k_B T}\right] \tag{S7}$$

where, D_0 is a constant diffusivity prefactor, Q_p, is the activation energy above the Néel temperature of pyrrhotite (588 K), α is a constant factor relating the magnetic ordering effect on the vacancy formation and migration energies, *S(T)* is the reduced magnetization relative to magnetization at zero Kelvin, k_B is the Boltzmann constant, and *T* is temperature. The values of D_0, Q_p, and α are given by Herbert et al. (2015)[4] are $D_0 = 4.65 \times 10^{-4}$ (cm²/sec), $Q_p = 8.3 \times 10^{-1}$ eV, and $\alpha = 0.41$. The plot of *S(T)* versus temperature from Herbert et al. (2015)[4] were used in the calculation. A previous measurement of iron diffusion at temperatures of >550 K reported a decrease in the diffusion coefficient of iron in $Fe_{1-x}S$ from pyrrhotite composition (x = 0.1) to almost troilite composition (x = 0.003) by about an order of magnitude[5]. We extrapolated D_{po} to lower temperatures and conservatively assumed $D_{tr} = (1/10)D_{po}$. We obtained a plot of *L* ($L = \sqrt{t_d D_{Tr}}$) versus temperature as shown in Supplementary Fig. 9, for the case where t_d = 1yr.

The temperature of the uppermost surface on Itokawa was estimated using a standard thermal model for an asteroid that has a spherical shape and zero thermal inertia. The assumption of zero thermal inertia is valid for uppermost surface of regolith grains (less than sub-micrometers depths), because the thermal skin depth of fine regolith; the depth where temperature decreases to 1/*e* of the diurnal temperature at mineral surface, is roughly estimated to be the order of a millimetre. The uppermost temperature was obtained by assuming thermal equilibrium between incoming solar power and radiative cooling as follows.

$$\epsilon\sigma T^4 = (1 - A)S/r^2 \quad \text{(S8)}$$

where ε denotes the bolometric emissivity of 0.125, σ is the Stefan-Boltzmann constant, T is the temperature at the subsolar point, A is the bolometric Bond albedo of 0.9, S is the solar constant, and r is the heliocentric distance in AU.

Assuming thermal equilibrium between incoming solar power and radiative cooling on the surface of Itokawa from the perihelion (0.953 AU, 401 K) to aphelion (1.69AU, 301 K), yields L values of 2.6×10^{-1} nm/yr to 1.32×10^{2} nm/yr. Given that present-day Itokawa spends ~10% of an orbital period within 1 AU (> 391 K), where L is larger than 77 nm/yr, the present-day average L on Itokawa' is the order of ten nm/yr. These estimates indicate that iron diffusion through the irradiation-damaged rims is faster than the production rate of excess iron, even if the damage microstructures are not taken into account, which would enhance Fe diffusion. Therefore, the excess iron atoms that are produced should rapidly diffuse in troilite.

Supplementary references

1. Enomoto, N., Muto, S., Tanabe, T., Davis, J. W. & Haasz, A. A. Grazing-incidence electron microscopy of surface blisters in single- and polycrystalline tungsten formed by H+, D+ and He+ irradiation. *J. Nucl. Mater.* **385**, 606–614 (2009).
2. Toulmin, P. & Barton, P. B. A thermodynamic study of pyrite and pyrrhotite. *Geochim.*

Cosmochim. Acta **28**, 641–671 (1964).

3. Nagao, K. *et al.* Irradiation History of Itokawa Regolith Material Deduced from Noble Gases in the Hayabusa Samples. *Science.* **333**, 1128–1131 (2011).

4. Herbert, F. W., Aravind, K., Lucy, R., Van Vliet, J. K. & Bilge, T. Magnetic diffusion anomaly at the Néel temperature of pyrrhotite, Fe 1−x S. *Phys. Chem. Chem. Phys.* **17**, 11036–11041 (2015).

5. Condit, R. H., Hobbins, R. R. & Birchenall, C. E. *Self-diffusion of iron and sulfur in ferrous sulfide*. *Oxidation of Metals* **8**, (1974).
